# Maximal expiratory pressure compared with maximal expiratory pressure during induced cough as a predictor of extubation failure

**DOI:** 10.5935/2965-2774.20230275-en

**Published:** 2023

**Authors:** Melina Carrera, Jose García Urrutia, Cesar Bueno Ardariz, Maria Luz Porra, Claudio Gamarra, Ladislao Pablo Diaz Ballve

**Affiliations:** 1 Hospital Nacional Profesor Alejandro Posadas - Buenos Aires, Argentina

**Keywords:** Airway extubation, Maximal respiratory pressures, Diagnostic techniques and procedures, Cough

## Abstract

**Objective:**

To compare the diagnostic performance of maximal expiratory pressure with maximal expiratory pressure during induced cough for predicting extubation failure within 72 hours in patients who completed a spontaneous breathing trial (SBT).

**Methods:**

The study was conducted between October 2018 and September 2019. All patients aged over 18 years admitted to the intensive care unit who required invasive mechanical ventilation for over 48 hours and successfully completed a spontaneous breathing trial were included. The maximal expiratory pressure was assessed with a unidirectional valve for 40 seconds, and verbal encouragement was given. The maximal expiratory pressure during induced cough was measured with slow instillation of 2mL of a 0.9% saline solution. The primary outcome variable was extubation failure.

**Results:**

Eighty patients were included, of which 43 (54%) were male. Twenty-two patients [27.5% (95%CI 18.9 - 38.1)] failed extubation within 72 hours. Differences were observed in the maximal expiratory pressure during induced cough between the group who failed extubation, with a median of 0cmH_2_O (P_25-75_: 0 - 90), and the group without extubation failure, with a median of 120cmH_2_O (P_25-75_: 73 - 120); p < 0.001.

**Conclusion:**

In patients who completed a spontaneous breathing trial, the maximal expiratory pressure during induced cough had a higher diagnostic performance for predicting extubation failure within 72 hours.

**Clinicaltrials.gov Registry:** NCT04356625

## INTRODUCTION

In recent years, several studies have identified different phases of the weaning process.^(1,2)^ Once a patient has successfully completed a spontaneous breathing trial (SBT) and no longer requires mechanical ventilation, it is reasonable to consider extubation. Intensivists are required to determine whether the patient can maintain a patent airway to reduce complications.

Although SBT is currently considered the best predictor of extubation failure and has a crucial role in the weaning phase,^(3)^ a successful SBT might not result in successful extubation (removal of the endotracheal tube).

Frutos-Vivar et al. observed that clinical indices, such as the rapid shallow breathing index (RSBI), were useful in predicting successful weaning from mechanical ventilation but not successful extubation.^(4)^ Similarly, Khamiees et al. found that successfully completing an SBT could not sufficiently predict extubation success.^(5)^

Extubation success is not often determined by passing an SBT. It is also necessary to evaluate whether patients can protect the airway by measuring the ability to cough effectively and assessing whether the cough is voluntary or a reflex. Cough can be voluntarily initiated and suppressed, but it can also be generated via the reflex pathway (controlled by the brainstem) that is only activated when the cough stimulus has reached the reflex threshold.^(6)^

The ability to cough was assessed in several ways, with different clinical variables, such as maximal expiratory pressure (MEP) and cough peak flow (CPF).^(7,8)^ The minimum values regularly used to predict extubation success ranged from 40 to 55cmH_2_O for the MEP^(9,10,11)^ and 29 to 160L/minute for the CPF.^(12,13,14)^

Patients with an altered state of consciousness cannot be assessed using these variables due to their inability to follow instructions. Su et al. assessed involuntary CPF (IV-CPF) induced by slow instillation of 2mL of a 0.9% saline solution and found that a value lower than 58.8L/minute was associated with extubation failure.^(15)^ Induced cough was also assessed in a study by Chan et al., who obtained a cutoff point of 29L/minute for IV-CPF in predicting successful decannulation of patients with neurological injury.^(14)^

To date, no studies have assessed MEP during induced cough (MEPic) as a method to predict extubation failure or have determined that MEP during cough is associated with a higher extubation failure rate. We hypothesize that MEPic has a higher diagnostic performance than MEP for predicting extubation failure within 72 hours.

Therefore, the objective of this study is to compare the diagnostic performance of the MEP with the MEPic for predicting extubation failure within 72 hours in patients who have successfully completed an SBT.

## METHODS

The protocol was approved by the Teaching and Research Committee and the Bioethics Committee of the hospital and registered at clinicaltrial.gov (NCT:04356625).

The study was conducted in a 26-bed polyvalent intensive care unit (ICU) of *Hospital Nacional Profesor Alejandro Posadas* between October 2018 and September 2019. The hospital is an acute general hospital that admits patients with medical and surgical pathologies. All patients aged over 18 years admitted to the ICU who required invasive mechanical ventilation (IMV) for over 48 hours and successfully completed an SBT according to the international consensus conference on weaning were included.^(1)^ Informed consent was obtained from all patients or their close relatives. Patients with a previous tracheostomy, neuromuscular disease history, unstable heart disease, upper gastrointestinal surgery, or untreated enterocutaneous fistula were excluded. Patients were also excluded if they were candidates for noninvasive mechanical ventilation as an interface switch for extubation or prevention, recruited in other studies, or unable to be assessed due to decompensation or procedure intolerance.

Due to the nature of the evaluation, neither the operators nor the patients could be blinded. The attending physician in charge of the decision to proceed or not with extubation was blinded to the MEP and MEPic values.

**Figure 1:**
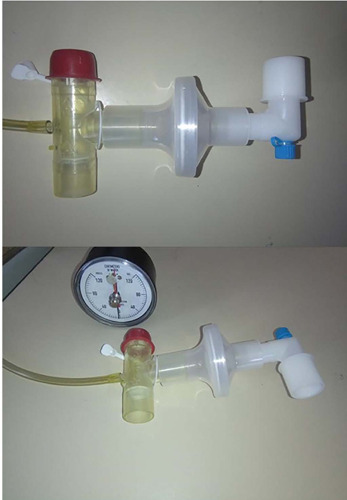
Settings for the measurement of maximal expiratory pressure during induced cough

Once the patient successfully completed the SBT in the supine position with the head of the bed elevated 45° - 60°, the outcome variables were measured. For this purpose, the closed suction catheter was removed, an elbow was positioned at 90°, and a bacterial filter was attached to the endotracheal tube. An adapter was also coupled with an outlet port to the aneroid pressure gauge, and an inspiratory unidirectional valve that did not allow expiration was attached, as shown in figure 1.

First, the MEP was assessed with the unidirectional valve for 40 seconds, and verbal encouragement was given using identical instructions to all patients. The patient was allowed to rest for 5 minutes. Then, the MEPic was measured with slow instillation of 2mL of a 0.9% saline solution through the port in the elbow at 90° to induce a cough (a similar stimulus to that created by secretions). During the procedure, special care was taken not to generate a cough when manipulating the endotracheal tube. The presence or absence of reflex cough and MEPic values were registered.

Maneuvers were stopped if the patient showed signs of intolerance, such as a change in safety variables (respiratory rate, heart rate, blood pressure, arterial O_2_ saturation) higher than 20% of baseline measures.

Sex, age, Acute Physiology and Chronic Health Evaluation II (APACHE II) score at admission, admission diagnosis, IMV duration, response to simple commands, semiquantitative cough strength score, and Glasgow coma scale score were analyzed. Once the patient successfully completed the SBT, the MEP and MEPic values in cmH_2_O and the presence or absence of reflex cough were registered.

The primary outcome variable was extubation failure, defined as the need for reinsertion of the endotracheal tube, the need for noninvasive mechanical ventilation as a rescue treatment, or death within 72 hours.

All data were collected on an encrypted database. The professional responsible for the statistical analysis was blinded.

### Statistical analysis

Numerical variables were presented using measures of central tendency and dispersion. Categorical variables are presented as numbers and percentages.

The incidence of extubation failure was presented as a proportion with the corresponding 95% confidence interval (95%CI).

The diagnostic features of the MEP and MEPic were determined for predicting extubation failure. Sensitivity, specificity, positive and negative predictive values, and positive and negative likelihood ratios with their respective 95%CIs were reported. The receiver operating characteristic (ROC) curve and Youden index were used to establish the optimal cutoff point for the MEP and MEPic for predicting extubation failure.

Outcome measures were compared in the subgroup of patients with neurological injury as the reason for ICU admission.

Statistical analysis, design, and graphs were performed using the R version 4.0 program.

A p value < 0.05 was considered statistically significant.

Sample size calculation was based on the area under the curve (AUC) of a similar predictor, which was 78% in a study by Su et al. A value of 50% was established as the null hypothesis with a difference in proportions between groups of 10%. The probability of a type I error was 5% with a power of 80%. The sample size was 80 patients.

## RESULTS

**Figure 2:**
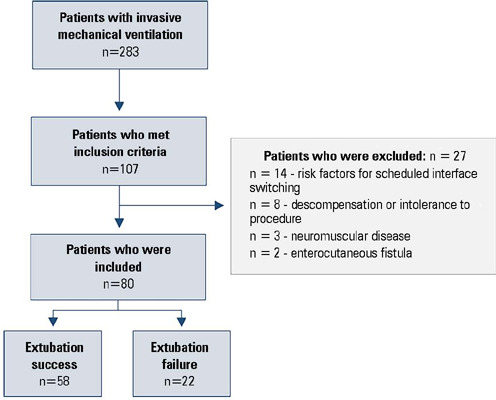
Study flowchart

Eighty patients were included (Figure 2), of which 43 (54%) were male. The mean age was 52 (standard deviation - SD ± 17.6) years. A total of 26 patients [32.5% (95%CI 23.2 - 43.3)] failed extubation within 7 days. Twenty-two patients [27.5% (95%CI 18.9 - 38.1)] failed extubation within 72 hours. The characteristics of the sample and comparisons between the group of patients with extubation success and the group with extubation failure are detailed in table 1.

**Table 1:** Characteristics of the sample

	Group without extubation failure within 72 hours n = 58	Group with extubation failure within 72 hours n =22	p value
Age	51.3 ± 17.8	54.0 ± 17.2	0.554*
Sex			
Female	25 (43.1)	12 (54.5)	0.453†
Male	33 (56.9)	10 (45.5)	
Medical reason for admission	29 (50.0)	13 (59.1)	0.617†
Surgical reason for admission	29 (50.0)	9 (40.9)	
Diagnostic group			
Neurological condition	18 (31.0)	5 (22.7)	
Respiratory failure	11 (19.0)	8 (36.4)	
PO abdomen	15 (25.9)	5 (22.7)	
PO head and neck	2 (3.4)	1 (4.5)	0.537‡
PO neurological	4 (6.9)	3 (13.6)	
PO trauma	2 (3.4)	0 (0.0)	
PO thorax	1 (1.7)	0 (0.0)	
PO others	5 (8.6)	0 (0)	
APACHE II	18.00 [11.0 - 23.0]	18.00 [15.0 - 24.5]	0.306§
IMV days	6.50 [4.2 - 11.00]	5.50 [4.0 - 11.5]	0.482§
GCS	15.00 [11.0 - 15.0]	15.00 [11.2 - 15.0]	0.910§
RSC #6	#0	2 (3.4)	0 (0.0)
#1	1 (1.7)	1 (4.5)	
#2	1 (1.7)	0 (0.0)	
#3	3 (5.2)	1 (4.5)	0.495‡
#4	2 (3.4)	3 (13.6)	
#5	5 (8.6)	3 (13.6)	
#6	44 (75.9)	14 (63.6)	
Simple commands_6	6.00 [6.0 - 6.0]	6.00 [5.0 - 6.0]	0.355 §
Presence of reflex cough	51 (87.9)	9 (40.9)	< 0.001†
SCSS			
No cough on command	8 (13.8)	4 18.2)	
Audible movement of air through the endotracheal tube but no audible cough	13 (22.4)	2 (9.1)	
Weakly (barely) audible cough	11 (19.0)	5 (22.7)	0.647‡
Clearly, audible cough	5 (8.6)	4 (18.2)	
Stronger cough	14 (24.1)	5 (22.7)	
Multiple sequential strong cough	7 (12.1)	2 (9.1)	
SBT			
CPAP	2 (3.4)	0 (0.0)	
PC-CSV	6 (10.3)	2 (9.1)	1.000‡
T-tube	50 (86.2)	20 (90.9)	
MEP	46.00 [40.0 - 80.0]	42.00 [34.25 - 51.5]	0.093§
MEPic	120.00 [73.0 - 120.0]	0.00 [0.00 - 90.0]	< 0.001§
Post-extubation cough			
No cough on command	11 (19.0)	3 (13.6)	
Effective cough	33 (56.9)	13 (59.1)	0.886‡
Ineffective cough	14 (24.1)	6 (27.3)	

PO - postoperative; APACHE II - Acute Physiology and Chronic Health Evaluation II; IMV - invasive mechanical ventilation; GCS - Glasgow coma scale; RSC #6 - response to 6 simple commands; SCSS - semiquantitative cough strength score; SBT - spontaneous breathing trial; CPAP - continuous positive airway pressure; PC-CSV - pressure control-continuous spontaneous ventilation; MEP - maximal expiratory pressure; MEPic - maximal expiratory pressure during induced cough. * Student’s t test; † chi-square test; ‡ proportion comparison test, with Bonferroni adjustment; §Mann-Whitney U test. Results expressed as mean ± standard deviation, n (%) or median (percentile 25 - percentile 75).

No significant differences were observed in the MEP values between the group of patients who failed extubation within 72 hours, with a median (Md) of 42 cmH_2_O (P_25-75_: 34.2 - 51.5), and the group without extubation failure, with an Md of 46cmH_2_O (P_25-75_: 40 - 80); p = 0.093. In contrast, differences were observed in the MEPic values between the group who failed extubation, with an Md of 0cmH_2_O (P_25-75_: 0 - 90), and the group without extubation failure, with an Md of 120cmH_2_O (P_25-75_: 73 - 120); p < 0.001. Nine patients who failed extubation [40.9% (95%CI 23.2 - 61.2)] presented reflex cough, whereas 51 patients without extubation failure [87.9% (95%CI 77.1 - 94.0)] presented reflex cough; p < 0.001.

**Figure 3:**
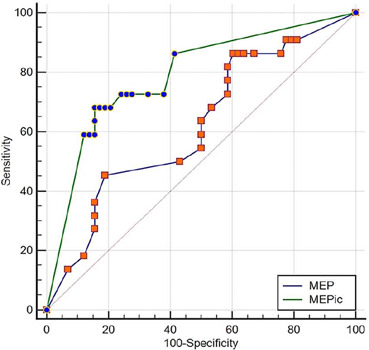
ROC curve comparison between the maximal expiratory pressure and maximal expiratory pressure during induced cough to predict extubation failure in the whole sample

The diagnostic properties of the MEP and MEPic are shown in table 2. The ROC curve analysis of the whole sample showed an AUC of 0.62 (95%CI 0.50 - 0.72) for the MEP and an AUC of 0.79 (95%CI 0.68 - 0.87) for the MEPic. The comparison between the two AUCs showed a difference of 0.17 (p = 0.01) (Figure 3).

**Table 2:** Diagnostic properties of the maximal expiratory pressure and maximal expiratory pressure during induced cough

	MEP 95%CI		MEPic 95%CI	
Criterion	≤ 38cmH_2_O	≤ 30 - ≤ 60	≤ 48cmH_2_O	≤ 0 - ≤ 100
Sensitivity (%)	45.4	24.4 - 67.8	68.1	45.1 - 86.1
Specificity (%)	81.0	68.6 - 90.1	84.4	72.6 - 92.7
Positive likelihood ratio	2.4	1.2 - 4.8	4.3	2.3 - 8.5
Negative likelihood ratio	0.67	0.5 - 1	0.38	0.2 - 0.7

MEP - maximal expiratory pressure; MEPic - maximal expiratory pressure during induced cough. Results expressed as %, when not otherwise indicated.

The area under the curve of maximal expiratory pressure was 0.62 (95%CI 0.50 - 0.72) and of maximal expiratory pressure during induced cough was 0.79 (95%CI 0.68 to 0.87), with a difference of 0.17 (p = 0.01).

MEP - maximal expiratory pressure; MEPic - maximal expiratory pressure during induced cough.

**Figure 4:**
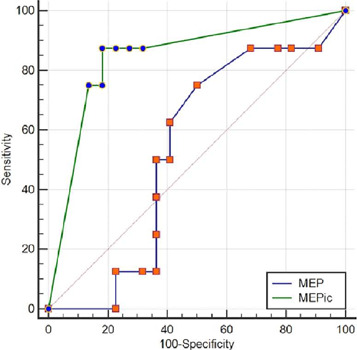
ROC curve comparison between the maximal expiratory pressure and maximal expiratory pressure during induced cough in the subgroup of patients with neurological injury

The analysis of the subgroup with neurological injury showed that 8 patients failed extubation [26.6% (95%CI 14.1 - 44.4)] within 72 hours. Differences were observed in the number of simple commands followed between the group of patients with extubation failure, with an Md of 4 commands (P_25-75_: 3.2 - 5), and the group without extubation failure, with an Md of 6 commands (P_25-75_: 4.7 - 6); p = 0.035. No differences were found in the MEP values between the two groups. Differences were found in the MEPic values between patients without extubation failure, with an Md of 120cmH_2_O (P_25-75_: 72 - 120), and patients who failed extubation, with an Md of 0cmH_2_O (P_25-75_: 0 - 120); p = 0.003 (Table 3). The ROC curves were compared and showed an AUC of 0.53 (95%CI 0.34 - 0.71) for the MEP and 0.84 (95%CI 0.66 - 0.95) for the MEPic. The comparison between the two AUCs showed a difference of 0.31 (p = 0.047) (Figure 4).

**Table 3:** Comparison in patients admitted for neurological reasons

	No failure n = 22	Failure n = 8	p value
Age	49.6 ± 16.5	54.6 ± 18.4	0.48 *
Female	9 (40.9)	5 (62.5)	0.41 †
APACHE II	17.6 (7.6)	21.4 (6.8)	0.25 *
GCS	13.5 (10.7 - 15)	12 (10.2 - 14.7)	0.5 ‡
SCSS	1.5 (0 - 4)	1.5 (0.2 - 3)	0.73 ‡
Simple commands (RSC #6)	6 (4.7 - 6)	4 (3.2 - 5)	0.03 ‡
IMV (days)	9.5 (5 - 14.2)	5 (3.2 - 11)	0.04 ‡
MEP	42 (37 - 90)	48.5 (41 - 58.7)	0.8 ‡
MEPic	120 (72 - 120)	0 (0 - 120)	0.003 ‡
Presence of reflex cough	18 (86.4)	2 (25)	0.003 †

APACHE II - Acute Physiology and Chronic Health Evaluation; GCS - Glasgow coma scale; SCSS - semiquantitative cough strength score; RSC #6 - response to 6 simple commands; IMV - invasive mechanical ventilation; MEP - maximal expiratory pressure; MEPic - maximal expiratory pressure during induced cough. * Student’s t test; † chi-square test; ‡ Mann-Whitney U test. Results expressed as mean ± standard deviation, n (%) or median (percentile 25 - percentile 75).

The area under the curve of maximal expiratory pressure was 0.53 (95%CI 0.344 - 0.71) and of maximal expiratory pressure during induced cough was 0.84 (95%CI 0.66 - 0.95), with a difference of 0.31 (p = 0.047).

## DISCUSSION

To our knowledge, this is the first study to evaluate MEPic as a method to predict extubation failure. The MEPic achieved an AUC of 80%, 17% higher than the MEP for predicting extubation failure within 72 hours. The cutoff point ≤ 48cmH_2_O for MEPic was associated with better sensitivity and specificity.

The MEP showed no differences, with a lower diagnostic performance predicting extubation failure within 72 hours. This is in line with a study by Vivier et al., which showed that the assessment of respiratory muscle functions using volumetric or pressure indices did not predict extubation failure.^(16)^ In contrast, Lai et al. observed that a cutoff point ≥ 55cmH_2_O for the MEP showed a positive predictive value of 95.8% for predicting successful extubation. According to the authors, the MEP is a useful indicator in predicting extubation success. However, the sensitivity and specificity ≥ 55cmH_2_O for the MEP showed poor predictive values (48% and 67%, respectively).^(9)^

Conversely, a study by Duan et al. showed that the diagnostic accuracy was significantly higher in voluntary CPF (V-CPF) compared with IV-CPF.^(17)^ The authors used the CPF as a cough substitute; however, we believe that the assessment of CPF is not accurate in patients with impairments in the compression phase due to laryngeal dysfunction, as the peak flow may underestimate the capacity of patients to protect the airway.

In the subgroup with neurological injury, the MEP correctly identified only 53% of patients who failed extubation, whereas the MEPic identified 84% of patients with extubation failure. Similarly, Duan et al. evaluated a similar population with a GCS < 13 and found that the IV-CPF was higher than the V-CPF. Despite the small sample size, the authors considered that the IV-CPF may be suitable for uncooperative patients.^(17)^ Similar results were reported in a study by Kutchak et al. in which it was concluded that the IV-CPF may be a predictor of successful extubation in neurological patients who were candidates for weaning from IMV.^(18)^

The absence of reflex cough was associated with extubation failure both in the entire sample and the subgroup with neurological injury. These results agree with those reported in a study by Godet et al.^(19)^

No differences were found in the state of consciousness, assessed with the GCS, between the groups in the entire sample, including the subgroup with neurological injury. These results are consistent with those found in previous studies.^(14,19)^ We believe that the state of consciousness assessed with the GCS is not useful for identifying patients with impaired reflex cough.

Finally, no differences in the semiquantitative cough strength score were observed between the groups. According to the author, patients with weak cough (grade 0 - 2) were four times more likely to have unsuccessful extubation, compared with patients with moderate-to-strong cough (grade 3 - 5) within 72 hours following extubation.^(5)^ In the subgroup with neurological injury, there was a significant difference in the ability to follow six simple commands between the groups, as opposed to the sample as a whole.

This study has some limitations. Due to the nature of the evaluation, operators and patients could not be blinded, which could have interfered with the values obtained. In addition, the sample size calculation was based on a similar AUC, as reported in a study by Su et al. In that study, the authors used the CPF to predict extubation failure. Due to the lack of similar studies, we could have made a type I error, i.e., an inappropriate sample size could have led us to reject the null hypothesis wrongfully. As a last limitation, the study was conducted in a single center. Therefore, further research is required to confirm or reject our findings.

## CONCLUSION

In patients who completed a spontaneous breathing trial, the maximal expiratory pressure during induced cough had a higher diagnostic performance for predicting extubation failure within 72 hours.
